# Differences in Cerebrospinal Fluid Biomarkers between Clinically Diagnosed Idiopathic Normal Pressure Hydrocephalus and Alzheimer’s Disease

**DOI:** 10.4172/2161-0460.1000150

**Published:** 2014-05-30

**Authors:** Andrew Tsai, Michael Malek-Ahmadi, Vickram Kahlon, Marwan N. Sabbagh

**Affiliations:** The Cleo Roberts Center for Clinical Research, Banner-Sun Health Research Institute, AZ, USA

**Keywords:** Cerebrospinal fluid, Alzheimer disease, Normal pressure hydrocephalus, Amyloid, Tau proteins, Diagnosis

## Abstract

**Objective:**

In the present study, cerebrospinal fluid (CSF) profiles were assessed to determine how idiopathic normal pressure hydrocephalus (NPH) and Alzheimer’s disease (AD) differs.

**Methods:**

Subjects were drawn from patients who underwent lumbar punctures as part of their diagnostic evaluations in the Banner Sun Health Research Institute Memory Disorders clinic. The clinical sample included 11 iNPH subjects (mean age 81.36±2.58) and 11 AD subjects (mean age 61.46±8.24). Concentrations of amyloid-β (Aβ42), total-tau (t-tau), phospho-tau181 (p-tau) Aβ42, and an Aβ42-Tau Index (ATI) were measured by commercial assay (Athena Diagnostics). and compared to each other. The Mann-Whitney test was used to assess group differences on the raw values for Aβ42, t-tau, p-tau, ATI, age, education, and MMSE.

**Results:**

In a univariate analysis, p-tau was found to be significantly (P = 0.009) lower in patients diagnosed with iNPH than those with AD. Amyloid-β (Aβ42), total-tau (t-tau) did not differ between groups. In multi-variate analysis, the differences in p-tau between groups did not differ.

**Conclusion:**

Although age could represent a significant confound, p-tau is significantly lower in iNPH compared to AD. P-tau would be expected to increase with age but in this sample is lower suggesting the difference might be explained by the underlying condition.

## Introduction

Alzheimer’s disease (AD) is characterized by a progressive dementia, the presence of neuritic plaques, and nerve cell degeneration [1]. Idiopathic normal pressure hydrocephalus (iNPH) was first described as a combination of gait disturbance, urinary incontinence, and cognitive impairment [2]. It is estimated that AD affects about 11% of individuals aged 65 years or older [3], whereas iNPH only affects 1.4% of the same population [4]. Of all patients clinically diagnosed with AD, post-mortem examinations reveal that 10%–20% of these patients have died with conditions other than AD [5]. Neuropathological examinations reveal that about 56% of patients clinically diagnosed with NPH also suffered AD [6].

Studies show that amyloid-β (Aβ42), total-tau (t-tau), and phospho-tau181 (p-tau) are effective biomarkers for AD patients [7]. The sensitivity and specificity for Aβ42 to discriminate AD from non-demented individuals is 86% and 89%, respectively; for t-tau, 81% and 91%, respectively; regarding p-tau, 81% and 91%, respectively; and for the combination of t-tau and Aβ42, 89% and 90% respectively [8]. However, these same biomarkers are much less understood when applied to iNPH, as results from studies are contradictory [9, 10]. Biomarkers are not routinely drawn for iNPH in clinical practice; incorporating these indicators may improve accuracy in the diagnosis of the dementia. Current methods for the diagnosis of iNPH include MRI scans, high volume cerebrospinal fluid (CSF) removal, nuclear medicine studies (cisternography), and evidence-based guidelines [11]. The only approved treatment for iNPH is a ventriculoperitoneal shunt [6]. There fails to currently exist a universally accepted system for classifying the impairment in each of the symptom triad domains for iNPH [11]. More studies are required to assess current biomarkers and discover new indicators of dementia, specifically for AD and iNPH.

The purpose of this study is to analyze CSF profiles to hopefully reveal differences between AD and iNPH. Continued studies on biomarkers could help reduce cases of misdiagnosis and also better understand the pathologies of AD and other types of dementia. In the present study, we test the hypothesis that the CSF profiles of individuals clinically diagnosed with iNPH and AD are different.

## Materials and Methods

### Subjects

The subjects were recruited from patients who underwent lumbar punctures as part of their diagnostic evaluation at the Banner Sun Health Research Institute memory disorder clinic. This study involved a retrospective chart review of CSF results for clinically diagnosed patients with iNPH and AD. Because of the retrospective nature of this study, an Institutional Review Board exemption was applied for and granted.

Included in the initial sample were 29 subjects. Only subjects clinically diagnosed with AD or iNPH were included. A total of seven subjects were excluded because they were diagnosed with a different type of dementia, specifically two with frontotemporal dementia (FTD), two for multiple sclerosis, one with multiple system atrophy, one with a connective tissue disease, and another with cognitive impairment involving depression. The final sample size was 22 and contained 11 NPH and 11 AD subjects.

### Diagnostic criteria

Clinical diagnoses of AD were based on NINCDS-ADRDA criteria [1] and iNPH using current evidence-based guidelines [12]. None of the subjects met clinical criteria for FTD according to the Lund and Manchester Groups criteria [13], Dementia with Lewy Bodies according to McKeith [14], or for vascular dementia [15].

### Lumbar puncture procedure

All lumbar punctures (LP) were performed after informed consent was obtained. Patients were prepped and draped in the usual sterile fashion. One percent lidocaine, without epinephrine, was used to achieve local anesthesia. The LP was done with a 20 gauge 3 inch spinal needle for both groups. CSF was collected and the needle was promptly withdrawn. The AD group had less than 12 cc drawn for diagnostic purposes. The NPH group underwent a higher volume lumbar puncture, drawing more than 25 cc. The CSF was then sent to Athena Diagnostics (Worcester MA) for laboratory tests for this study.

### CSF measurement

The concentrations of Aβ42, tau and p-tau in CSF were determined by ELISA tests specifically made by Innogenetics NV (InnotestTMβ- Amyloid(1–42), InnotestTMhTAU Ag, and InnotestTMPhospho-tau). Calculated amounts are based on standard curves using synthetic Aβ42, tau, and a synthetic τp-181. An Aβ42 Tau Index was calculated from the formula: ATI = (Aβ42)/(240+1.18*(tau)) (Athena Diagnostics) [16, 17].

### Statistical Analysis

The Fisher exact test was used to assess differences in gender frequency between the AD and NPH groups. The Mann-Whitney test was used to assess group differences on the raw values for Aβ42, t-tau, p-tau, ATI, age, education, and MMSE. Raw values for Aβ42, t-tau, p-tau, and ATI were log-transformed in order to normalize the distributions. The group differences of the log-transformed values were then analyzed using an analysis of covariance (ANCOVA) using age, education, and gender as covariates in order to account for their effect. Geometric means with 95% confidence intervals are reported for the log-transformed values. Spearman correlation analysis was used to analyze the association between age and p-tau levels.

## Results

The 22 cases used for this sample were divided evenly between the AD and iNPH groups. For the entire sample, there were 9 males and 13 females. Age, MMSE score, gender distribution, and education level for the two groups are displayed in Table 1. MMSE scores and years of education were not significantly different between the two groups. The iNPH group was significantly older than AD group. There was no significant association for gender and diagnosis.

Analyses of the raw values for ATI, Aβ42, t-tau, and p-tau concentrations were measured and shown in Table 1 and in the figure. CSF p-tau which was significantly lower in the iNPH group in an unadjusted analysis. Analyses of the CSF markers using the log-transformed values showed no significant difference for any of the markers after adjusting for age, education, and gender. There was an inverse relationship between age and p-tau levels as p-tau levels tended to increase with younger ages (r = −0.48, p = 0.02).

## Discussion

In this retrospective study involving CSF profiles of subjects clinically diagnosed with AD or iNPH, we found the p-tau concentrations to be significantly lower in iNPH suggesting different CSF profiles between the two groups. Though the p-tau is lower in iNPH in univariate analysis, the observation is less apparent in multivariate analysis. MMSE scores, education, Aβ42, total tau, and ATI showed no significant differences.

Prior studies have investigated the use of CSF as a means to differentiate AD from other dementias. They have shown that CSF profiles can separate AD from FTD, LBD, and Creutzfeldt-Jakob disease (CJD) [18–21]. In one study, using a t-tau by Aβ42 plot, it was possible to discriminate AD and non-AD areas of the plot, resulting with 92% of the AD subjects within the AD area [11]. It was also reported that tau was an effective biomarker for CJD; its sensitivity and specificity was 94% and 90%, respectively, with a positive predictive value of 92% [19]. P-tau also showed a sensitivity of 72% and specificity of 93% when used to differentiate early-onset AD and FTD [20]. These studies show that analyzing CSF profiles can be a powerful approach to differentiating dementias.

One strength of this study is that it utilized a side by side comparison of clinically diagnosed cases that were prospectively assessed using established clinical criteria, allowing differences to be more easily observed. However, two significant weaknesses are apparent. First, the small sample size of the NPH and AD groups may hinder interpreting the results on a larger scale. Second, is the groups in the study are not age-matched. This likely reflects selection bias of the cases. The iNPH were selected strictly on their clinical criteria and CSF was acquired as part of the diagnostic evaluation. However, the AD group underwent lumbar punctures for diagnostic confirmation because they had a pre-senile presentation of the disease.

In our study, we also find that age is an important co-variate of p-tau. Paradoxically, it appears to go down with age in AD and iNPH. Since the iNPH subjects are significantly older than the AD subjects, we would expect the higher p-tau in the AD group to be attributed to age. Paternico *et al.* [22] identified that the same CSF biomarkers of AD investigated here were associated with age in cognitively normal young and old. In their study, p-tau increases with age. Thus, the difference in the p-tau levels observed between the groups is more likely related to the underlying pathology than the age differences alone.

Kapaki *et al.* [18] have analyzed the CSF profiles of AD, iNPH, and control groups. However, their methods differed as they compared iNPH and AD to a control group, whereas AD and NPH are compared to only each other in this study. Despite differences in comparison methods, many of our results are in agreement. For example, p-tau was found to be elevated for AD in both studies. Aβ42 levels were lowered in both AD and iNPH, which support our finding that Aβ42 concentrations are not different between the two groups. They also found that total tau was significantly increased in iNPH and highly increased in AD when compared to controls while the difference in total tau was not significantly different in the current study.

Sjörgen *et al.* [21] attempted to establish reference values for CSF profiles and analyzed effects of age and gender on CSF. They found a correlation between age and t-tau, and thus created values for separate age groups. For t-tau, they reported<399ng/L for ages 21–50, <450 ng/L for ages 51–70, and <500 ng/L for ages 71–90. The average ages for our AD group were 61 and NPH was 81. According to their reference values for t-tau, there should be a difference of 50 ng/L between the two groups that our samples fall under. Regarding gender effects on CSF, they found no significant differences in t-tau and AB42 between men and women.

The average total protein concentrations for the AD and NPH group were 42.57 mg/dL and 53.25 mg/dL, respectively. The average total protein levels of the two groups fall under the normal range for healthy individuals, which range from 15–60 mg/dL [23].

It has been proposed that analyzing CSF may become an effective method in differentiating NPH and AD [10]. Further studies with larger sample sizes are required to assess the value of biomarkers when used to differentiate between AD and NPH. The findings in this study may guide others to better study the uses of CSF analysis as a tool in the diagnosis of iNPH.

## Figures and Tables

**Figure:**
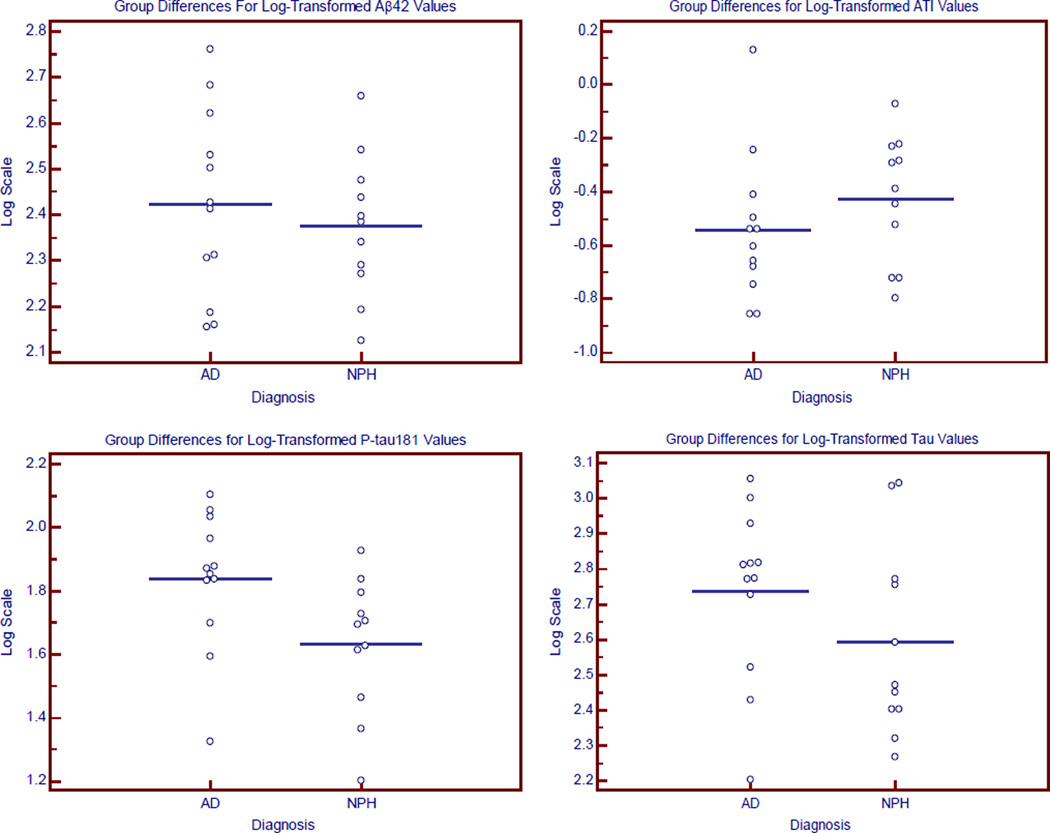
The CSF profiles of AD and iNPH.

**Table 1:** Demographic and Clinical Characteristics.

	AD	NPH	p-value
N	11	11	------
Males/Females (n)	2/9	7/4	0.47
Age	61.46±8.24	81.36±2.58	<0.001
Education	14.55±2.70	15.13±3.52	0.93
MMSE	18.89±8.68	19.13±8.32	0.92
Aβ 1-42 (pg/ml)†	290.33±146.74	251.17±92.39	0.72
Tau (pg/ml)†	651.53±276.41	475.07±335.23	0.08
P-tau 181 (pg/ml)†	79.12±30.00	47.35±20.24	0.009
ATI †	0.34±0.34	0.43±0.21	0.11
Aβ 1-42 (pg/ml)‡	260.02 (187.07, 361.41)*	237.14 (186.64, 300.61)*	0.72^1^
Tau (pg/ml)‡	584.79 (406.44, 839.46)*	391.74 (256.45, 597.04)*	0.84^1^
P-tau 181 (pg/ml)‡	72.44 (52.12, 100.46)*	42.85 (30.76, 59.70)*	0.33^1^
ATI‡	0.27 (0.18, 0.41)*	0.37 (0.26, 0.54)*	0.57^1^

mean±standard deviation;

† raw scores;

‡ log-transformed scores;

* geometric mean (95% confidence interval);

1 adjusted for age, gender, and education
